# Variability of activity patterns across mood disorders and time of day

**DOI:** 10.1186/s12888-017-1574-x

**Published:** 2017-12-19

**Authors:** Karoline Krane-Gartiser, Arne E. Vaaler, Ole Bernt Fasmer, Kjetil Sørensen, Gunnar Morken, Jan Scott

**Affiliations:** 1 Department of Mental Health, NTNU, Norwegian University of Science and Technology, Trondheim, Norway; 2 Department of Psychiatry, St. Olav’s University Hospital, Trondheim, Norway; 3 Department of Clinical Medicine, Section for Psychiatry, Faculty of Medicine and Dentistry, University of Bergen, Bergen, Norway; 4 Division of Psychiatry, Haukeland University Hospital, Bergen, Norway; 5 Academic Psychiatry, Institute of Neuroscience, Newcastle University, Newcastle Upon Tyne, UK

**Keywords:** Unipolar, Bipolar, Mixed states, Actigraphy, Motor activity, Time series, Variability, Non-linear analysis

## Abstract

**Background:**

Few actigraphy studies in mood disorders have simultaneously included unipolar (UP) and bipolar (BD) depression or BD mixed states as a separate subgroup from mania. This study compared objectively measured activity in UP, BD depression, mania and mixed states and examined if patterns differed according to time of day and/or diagnostic group.

**Methods:**

Eighty -eight acutely admitted inpatients with mood disorders (52 UP; 18 mania; 12 BD depression; 6 mixed states) underwent 24 hours of actigraphy monitoring. Non-parametric analyses were used to compare median activity level over 24 h (counts per minute), two time series (64-min periods of continuous motor activity) in the morning and evening, and variability in activity across and within groups.

**Results:**

There was no between-group difference in 24-h median level of activity, but significant differences emerged between BD depression compared to mania in the active morning period, and between UP and mania and mixed states in the active evening period. Within-group analyses revealed that UP cases showed several significant changes between morning and evening activity, with fewer changes in the BD groups.

**Conclusions:**

Mean activity over 24 hours has limited utility in differentiating UP and BD. In contrast, analysis of non-linear variability measures of activity at different times of day could help objectively distinguish between mood disorder subgroups.

**Trial registration:**

ClinicalTrials.gov Identifier: NCT01415323, first registration July 6, 2011.

## Background

New technologies are increasingly applied to monitor a range of symptoms observed in mood disorders [1]. Actigraphy is now a recognized tool for measuring rest-activity patterns that provides objective and ecologically valid data about motor activity over 24 h [2]. Until recently, actigraphy has been used to assess sleep profiles in bipolar disorders (BD) [3, 4], but now there has been a shift in attention towards an examination of daytime activity and the 24-h rest-activity cycle in unipolar depression (UP) and BD [5, 6]. This change in focus coincides with the modification of diagnostic criteria for BD described in the 5th edition of the Diagnostic and Statistical Manual of Mental Disorders (DSM-5) [7] and its revision of the “criterion A symptom” for hypomania and mania, so that it now includes disturbed energy or activity alongside change in mood state as a key element of the diagnosis. While this revision has been welcomed by many researchers, it is noteworthy that the DSM-5 fails to indicate whether disturbed activity may also be a major criterion of bipolar depression (BD depression), whether it can distinguish BD depression from UP, what part it plays in mixed states, and critically, in mania and hypomania, it remains unclear whether the observed change in activity refers to mean level, predictability, or variability, etc. [6].

The application of actigraphy to measure daytime activity as well as sleep patterns represents an important opportunity for research in mood disorders and for instance, it may help confirm or refute whether reduced motor activity is a more common feature of BD depression compared to UP (as suggested in some clinical studies) and/or whether the overall patterns of activity differ over 24 h (possibly underlying circadian disturbances) [8, 9]. However, actigraphy studies of motor activity have usually been conducted in either UP or BD [5, 10–12], with comparator groups usually comprised of healthy controls (for a review, see [6]). To date, few studies have compared UP and BD groups that were recruited at the same time and/or from the same setting [13–17].

As well as a limited number of direct comparisons between UP and BD, many actigraphy studies have restricted the analyses to basic parameters representing the sleep-wake cycle (duration of sleep, sleep latency, mean activity levels, etc.) Findings from recent studies and a systematic review suggest that mean levels of activity alone may be less useful for distinguishing between different subtypes of mood disorders than an exploration of variability, predictability or complexity of activity patterns [6, 18–20]. This is reinforced by our own recent studies [21–23]. For example, the study of acutely admitted BD cases (with mania and depression), found that the most noticeable differences between BD and healthy controls were better described by the variability and complexity of the actigraphy recordings, rather than by mean activity levels [21]. Even a study of UP cases classified as having motor-retarded and non-motor retarded depression [22] highlighted that, as well as lower mean activity levels in retarded depression, these cases also showed higher intra-individual variability in activity over 24 h than non-retarded depression. Furthermore, a time series analysis of an active morning period demonstrated that UP cases without motor retardation displayed significantly increased complexity or disorder in their activity patterns compared to motor-retarded cases. These studies, along with others that used non-linear analytic strategies, suggest that depression may be characterized by an increase in intra-individual variability in activity with patterns retaining a certain regularity [19, 21, 22, 24], whereas manic cases have shown more disorganized activity patterns, but not necessarily an increase in mean level of activity [6, 21].

The methodologies used in these recent studies and the insights provided have helped to develop our strategy for mathematical analyses of activity patterns in UP and BD. They also reveal that there are several key questions that remain unanswered. First, few studies compare different patterns of activity in cases of UP to BD depression, and secondly, there are hardly any actigraphy studies that examine separately cases presenting with a mixed state. The latter is a less frequent presentation of BD, and these cases are usually omitted from actigraphy studies or subsumed within groups presenting with mania [25]. Lastly, our former studies indicate that time series analysis of BD depression and mania or different subtypes of UP demonstrate some differences in activity within these mood disorder groups during selected active morning or evening periods. However, no direct comparison of UP and BD cases is available. Also, it is not clear how activity parameters measured in the morning and evening differ within mood disorder subtypes, and whether a difference would be seen only in overall activity level or also in variability or regularity of patterns.

To answer these unresolved questions, we undertook a preliminary pilot study to explore if four ICD-10 diagnostic subgroups with mood disorders (UP and BD depression, mania and mixed states) differed in measures of activity, and whether there were within-group differences in activity as measured in the morning compared to the evening. We recruited cases presenting to the same clinical setting (namely an acute admission unit), and selected a priori four variables that our previous research indicated may best characterize key, but different aspects of activity and that were intuitively comprehensible to clinicians. These were: median level of activity and three different measures of variability (fluctuations from the mean, minute-to-minute differences and regularity of the pattern). Given the constraints of the study (as we predicted that group sizes would be uneven and some groups would be small), the most important goal was to determine if the methodology and strategy may have utility for larger scale studies.

## Methods

### Sample

The study methodology is detailed in the previous publications [21, 22], but can be summarized as follows: individuals aged > 18 years with a confirmed diagnosis of UP or BD, who were acutely admitted as inpatients at the Department of Psychiatry, St. Olav’s University Hospital, Trondheim, Norway, between September 1^st^, 2011 and March 31^st^, 2012 and who were willing and able to give written informed consent, were asked to participate in an ethically approved assessment study that included recording of their sleep-wake activity patterns for 24 h during one of the earliest days of their admission.

Diagnosis was made according to ICD-10 “Criteria for research” (WHO, 1993) by clinicians with experience of the diagnosis and treatment of mood disorders and the application of international diagnostic criteria. Three specialists of whom at least two knew the patient from the inpatient stay, set the diagnosis in a consensus meeting at discharge, using all available information. The diagnostic subgroups included a primary diagnosis of a depressive episode or recurrent depressive disorder (F32 and F33), a primary diagnosis of BD, current episode manic (F31.1-F31.2), a primary diagnosis of BD, current episode depressed (F31.3-F31.5) or a primary diagnosis of BD, current episode mixed (F31.6).

### Actigraphy

- *Recordings of Motor Activity*

All recordings used a wrist-worn actigraph (Actiwatch Spectrum, Philips Respironics Inc., Murrysville, USA), and activity counts per minute were recorded over 24 h. Monitoring began during daytime hours (mean approximately 12:30 PM; SD approximately 2.5 h).

As noted in our previous actigraphy studies [21, 22] we analyzed the full 24-h period. In addition, we selected actigraphy data from 6 AM to midnight for each case and separated this time period into morning and evening sequences. Morning sequences were defined to occur between 6 AM and 3 PM, and evening sequences between 3 PM and midnight. Next, we searched each morning and evening sequence for periods of continuous motor activity, because periods containing immobility would distort the results of certain analytic approaches. The active morning period was searched from the start of the series, and the active evening period from the end of the series. In order to obtain the first and last period of activity, adapted to each individual’s circadian rhythm and not at a specific point in time, we selected the first period of 64 min not containing more than two consecutive minutes of zero activity counts for each case. If there was no such period, we searched for sequences with no more than 3 or 4 consecutive minutes of zero activity. We were unable to obtain 64-min sequences for the morning and/or the evening for four cases (two with UP and two with mania), so these cases were omitted from the analyses of active periods. In the experience of the authors, it can be difficult to find periods of continuous activity that are longer than approximately one hour in inpatients, particularly in depressed individuals. The reason for choosing 64 min instead of 60 min, is that one of the analyses in previous papers, the Fourier analysis, requires time series to be potencies of 2 (32, 64, 128 min, etc.) [21].

b) *Activity Parameters*

From the activity counts in the actigraph software program (Actiware, version 5.70.1) we calculated median activity counts per minute for the whole 24-h recording period. For the 64-min periods of continuous activity in the morning and evening, we calculated:

1. The median activity count per minute, as a measure of overall activity level.
2. The standard deviation (SD) for each time series, as an intra-individual measure of fluctuations in activity.
3. The root mean squared successive difference (RMSSD, which describes the difference in successive counts from minute to minute), as a measure of intra-individual variability in the time series.
4. Sample entropy, as a non-linear measure of the degree of regularity (complexity) of a time series.

Sample entropy was used as it can be employed with short time series of minimum 50 data points and is robust regarding outliers [26]. Sample entropy is the negative natural logarithm of an estimate of the conditional probability that subseries of a certain length (m) that match point-wise, within a tolerance (r), also match at the next point, meaning that a high entropy value indicates high complexity or a more irregular time series. The data were normalized by transforming the sequence to have sample mean 0 and sample variance 1. Sample entropy was estimated using open access software that is available online (the Physio Toolkit; http://www.physionet.org), and we chose the following values for the analysis: m = 2 and *r* = 0.2. SD and RMSSD are given as values in percent of mean activity, in order to account for the level of activity and allow comparison between individuals.

### Statistics

We used SPSS (version 22) for all the reported statistical analyses. All tests were non-parametric, with statistical significance set a priori at *p* < 0.5. We did not apply any corrections for multiple testing, as we undertook only a limited range of pre-selected analyses and also because we wished to identify trends across groups or within groups across time.

The analyses proceeded in three steps:

- Description of the sample: gender distribution (Pearson’s chi-squared test), median age at study inclusion (and inter-quartile range (IQR) and median activity levels over the 24-h monitoring period (Kruskal-Wallis tests) were compared between diagnostic subgroups.
- Between-group comparisons: the primary analyses of differences in the morning and evening activity patterns were evaluated using Kruskal-Wallis tests, and we report medians and inter-quartile ranges (25^th^ and 75^th^ percentiles) for each parameter at each time point. Also, we undertook pairwise comparisons between diagnostic subgroups using Mann-Whitney U tests.
- Within-group comparisons: We used Wilcoxon signed-rank tests for the exploration of within-group differences in activity patterns in the selected morning and evening periods and report the paired median difference for each parameter by group.

## Results

The diagnostic subgroups were distributed as follows: 52 patients were admitted with a primary diagnosis of a depressive episode or recurrent depressive disorder (F32 and F33), 18 patients with a primary diagnosis of BD, current episode manic (F31.1-F31.2), 12 patients with a primary diagnosis of BD, current episode depressed (F31.3-F31.5) and finally, 6 patients with a primary diagnosis of BD, current episode mixed (F31.6). The sample median age was 40 years, and there were 47 females and 41 males (see Table 1). The median activity count per minute (undertaken over 24 h) did not differentiate between diagnostic subgroups. The medication categories across subgroups are shown in Table 2.

**Table 1:** Basic characteristics of 88 cases who were admitted to a psychiatric department with an acute mood episode: gender distribution, age and 24-h activity count

Diagnosis	Number of Females: Males	Median (Inter-Quartile Range)	
		Age in years	Activity count per minute (24 h)^a^
Unipolar Depression	26:26	40 (30, 52)	95 (74, 132)
Bipolar Depression	7:5	35 (27, 56)	113 (80, 175)
Mania	11:7	50 (42, 67)	140 (111, 193)
Mixed States	3:3	41 (28, 57)	102 (83, 191)
Chi-squared^b^	.82	5.32	7.17
Significance (df = 3)	.845	.150	.067

^a^The activity count is calculated per minute from the full 24-h recording

^b^Pearson chi-squared test used for analysis of gender distribution; Kruskal-Wallis test used for analysis of age and activity counts

**Table 2:** Medication categories across diagnostic subgroups

Medication category	Unipolar depression (*n* = 52)	Bipolar depression (*n* = 12)	Mania (*n* = 18)	Mixed states (*n* = 5)^a^
Antipsychotics	9 (17)	5 (42)	15 (83)	2 (40)
Hypnotics/anxiolytics	22 (42)	6 (50)	9 (50)	2 (40)
Anticonvulsants	8 (15)	4 (33)	7 (39)	4 (80)
Lithium	0	1 (8)	2 (10)	0
Antidepressants	22 (42)	1 (8)	1 (5)	0
ECT	1 (2)	0	0	0
No psychotropic drug treatment	9 (17)	1 (8)	1 (5)	1 (20)

^a^Medication information was available for 5 of 6 patients in the mixed states subgroup

All values are given as n (%)

The analysis of the active morning period did not reveal any significant differences in median level or minute-to-minute variability in activity (as measured by the RMSSD) according to diagnostic subgroups. However, as shown in Table 3, there were significant differences in intra-individual fluctuations (as measured by the SD; *p* = 0.030) and complexity of activity (as measured by the sample entropy; *p* = 0.036). Pairwise comparisons demonstrated that BD depression showed a higher SD than mania (*p* = 0.033). In contrast, in the evening period pairwise comparisons revealed that cases in a mixed state had significantly higher levels of activity in the evening than UP cases (*p* = 0.019), but the overall between-group analysis of median activity count did not demonstrate any significant differences (Kruskal-Wallis test, *p* = 0.068). Also, there was a significant difference in intra-individual fluctuations in activity (SD) across the diagnostic subgroups (*p* = 0.039), with pairwise comparisons highlighting that UP had a significantly higher SD than mania (*p* = 0.005).

**Table 3:** Comparison of the active morning and evening periods across diagnostic subgroups

Median & Inter-Quartile Range (25^th^ & 75^th^ percentiles)		ACTIVE MORNING PERIOD				ACTIVE EVENING PERIOD			
		Activity count per minute	SD in % of activity count	RMSSD in % of activity	Sample entropy	Activity count per minute	SD in % of activity count	RMSSD in % of activity count	Sample entropy
Unipolar Depression (*n* = 50)	Median	185	103.3	92.0	1.066	152	114.8	113.2	0.940
	25^th^	124	88.5	73.6	0.667	84	97.0	85.3	0.519
	75^th^	325	130.1	116.0	1.399	217	153.3	142.8	1.373
Bipolar Depression (*n* = 12)	Median	245	102.4	96.2	0.976	201	108.4	102.5	1.054
	25^th^	168	91.0	82.2	0.586	98	91.2	88.2	0.852
	75^th^	310	130.0	114.2	1.472	238	149.4	127.9	1.285
Mania (*n* = 16)	Median	169	86.7	81.8	1.353	187	88.1	83.9	1.090
	25^th^	110	64.6	63.1	1.129	132	72.3	73.2	0.678
	75^th^	374	109.4	111.5	1.712	281	113.2	112.8	1.742
Mixed States (*n* = 6)	Median	227	94.2	90.4	1.405	279	106.7	88.8	0.904
	25^th^	159	67.8	53.8	1.202	176	87.3	81.4	0.758
	75th	508	101.5	113.7	1.788	363	133.9	108.2	1.157
Kruskal-Wallis Test	Chi-squared	1.52	8.94	1.90	8.54	7.14	8.35	6.24	3.19
	Significance (df = 3)	.679	.030	.593	.036	.068	.039	.100	.363
Pairwise comparisons (Mann-Whitney U tests)			*M vs. BDD * *(p = 0.033)*MS vs. BDD (*p* = 0.151)		M vs. BDD (*p* = 0.110)MS vs. BDD (*P* = 0.102)	*MS vs. UP* *(p = 0.019)*M vs. UP (*p* = 0.110)	*M vs. UP* *(p = 0.005)*		

*SD* Standard Deviation, *RMSSD* Root Mean Square of Successive Differences, *BDD* Bipolar Depression; *M* Mania, *MS*, Mixed States, *UP* Unipolar Depression

As shown in Table 4, the within-group comparison of active morning and active evening periods revealed that UP cases showed significant differences in median activity (counts per minute decreased in the evening; *p* < 0.001), intra-individual fluctuations (SD higher in the evening; *p* = 0.019) and variability (RMSSD higher in the evening; *p* < 0.001). For the BD groups, mania cases showed no significant changes between morning and evening. In BD depression cases, median activity was lower in the evening (*p* = 0.019), while cases admitted in a mixed state showed an increase in intra-individual fluctuations (SD; *p* = 0.046).

**Table 4:** Comparison of the active morning and evening periods within diagnostic subgroups

DIAGNOSIS	ACTIVE EVENING VS. MORNING PERIODS	Wilcoxon signed-rank test		
		Median difference	z statistic	Significance**
Unipolar Depression	Mean activity count per minute	−65	3.51	< 0.001
	SD* in % of mean activity count	11.82	2.35	0.019
	RMSSD* in % of mean activity	15.02	3.51	< 0.001
	Sample entropy	−0.059	1.07	0.284
Bipolar Depression	Mean activity count per minute	−31	2.35	0.019
	SD* in % of mean activity count	6.43	0.94	0.347
	RMSSD* in % of mean activity	8.20	1.02	0.308
	Sample entropy	0.083	0.47	0.638
Mania	Mean activity count per minute	0	0.06	0.955
	SD* in % of mean activity count	0.27	0.74	0.460
	RMSSD* in % of mean activity	2.37	0.85	0.394
	Sample entropy	−0.325	0.72	0.469
Mixed State	Mean activity count per minute	−40	0.31	0.753
	SD* in % of mean activity count	29.17	1.99	0.046
	RMSSD* in % of mean activity	17.76	0.94	0.345
	Sample entropy	−0.634	1.57	0.116

^*^ *SD* Standard Deviation, *RMSSD* Root Mean Square of Successive Differences

^**^degrees of freedom by group: Unipolar Depression = 47; Bipolar Depression = 11; Mania = 15; Mixed States = 5

## Discussion

This exploratory study considers how mood disorder subgroups in an acute state differ from each other in terms of activity patterns measured continuously over 24 h and for selected 64-min time periods in the morning and evening. The findings confirm that mean activity levels alone do not distinguish phase of illness in BD (BD depression, mania or mixed state), nor subtype of illness in mood disorders (BD or UP), but that detailed time series analysis of active morning and evening periods may reveal differences between diagnostic subgroups or differences within the same subgroup over time. The latter may help to illuminate underlying mechanisms of changes in activity within subgroups or specific causes of any objectively measured changes, e.g. weakening or loss of circadian rhythmicity.

This pilot study identified three main findings. First, in BD, the BD depression and mania subgroups differ in activity patterns in a selected active morning period, with significantly increased intra-individual fluctuations (SD) and a trend for more regular patterns (lower sample entropy values) in BD depression. In contrast, measures of evening activity did not differentiate between the BD subgroups. Second, the most overt differences in the active evening period were between UP and different BD subgroups. Notably, UP differed from mania in intra-individual fluctuations in activity, and UP differed from mixed states in median activity. Third, within-group analyses suggested that UP cases showed the most significant change in activity profiles from morning to evening, with a significant decrease in median activity and a significant increase in intra-individual fluctuations (SD) and minute-to-minute variability (RMSSD) in the evening. There were few distinct changes between the active morning and evening periods within the three BD subgroups. It is notable that mania cases showed no evident difference over time in any of the variables assessed.

There were no significant differences between BD depression and UP in mean activity levels or variability measures, neither in the morning nor in the evening. Previous studies that have compared UP to BD depression have found lower activity levels in BD depression cases than UP cases [14–16]. We could not replicate this finding; the BD depression group in the current study had higher median levels than UP both during 24 h and in the active morning and evening periods. However, the current study used two specified, brief time periods that gave a shorter “snapshot” of activity. It further included acutely hospitalized and medicated inpatients at an early stage of their admission and also the sample sizes, stability of symptoms and/or severity of the depressive episode varied between the studies. Methodologically, the difference in medication status and the fact that we extracted a period of approximately one hour with continuous motor activity (meaning that periods of immobility were excluded) may well explain some of the differences in the findings. For this reason, our study cannot address potential differences in distribution of reduced motor activity between UP and BD depression, which observational studies traditionally considered a more common feature of BD depression [8]. It is probable that depressive subgroups are more heterogeneous in terms of motor activity and that differences between UP and BD depression subgroups might be exposed if the depressive presentations were categorized according to the severity of symptoms, loss of diurnal variation, and/or clinical presentation of agitation or retardation. This would, of course, require an even larger sample and additional stratification of cases, along with more intensive clinical assessments. While this is worth considering in future studies, it must be borne in mind that the requirements of the research protocol must be balanced against participant burden and the clinical needs of acutely unwell patients. Until the findings of such studies are available, our study highlights that activity, as a criterion of depression seems to be insufficiently explored within our current diagnostic framework and more attention is merited to provide more solidly substantiated diagnostic criteria.

Although our findings further suggest that acute UP and BD depression cannot be told apart by comparing activity variables in a specific time series, the groups themselves may differ in the activity profiles seen within each subgroup at different times of day. As an example, the UP group displayed more differences in activity patterns between the morning and evening than the BD depression group. Considering the large discrepancy in size (50 vs. 12 subjects), the findings should be interpreted with caution. However, the relative diurnal stability seen in the BD groups in our study agrees with recent papers suggesting that severity of manic symptoms in BD may be correlated with circadian dysrhythmia [18] and that BD is associated with lower circadian rhythmicity compared to UP in young adults [13]. Likewise, an ongoing study of nearly 500 participants who are monitored with actigraphy for 2 weeks, reported that individuals with BD demonstrated a disturbance of sleep and activity with greater variability and circadian shifts compared to major depression and anxiety disorders [17]. Taken together, these studies add weight to our finding of differences in intra-daily stability of activity characteristics in BD compared to UP.

Manic and mixed states showed a tendency of increased median activity levels from morning to evening, which is consistent with some but not all previous findings [6] and may imply a phase-delay in peak of activity [25]. The manic cases also demonstrated stable patterns in terms of variability measures from morning to evening in the within-group comparison. While these results need to be verified in larger samples, they may indicate that there is a more significant diurnal variation in UP than BD. We were keen to include a subgroup of cases in a mixed state, even though the group was small, as we wanted to explore which activity patterns may occur more often in mixed states and to consider whether they are more similar to depressive or manic patterns. In the current investigation, cases admitted with a mixed state displayed activity patterns that were similar to profiles seen in the manic cases, particularly as demonstrated by low variability and high entropy values, hinting at the higher degree of disorder found in both these phases of BD. However, the intra-individual fluctuations increased from morning to evening in mixed states, signaling a possible diurnal change and also reflecting the findings in the UP group. Consequently, in a larger sample it could be hypothesized that activation in mixed states resembles mania in the morning, but to a greater degree produces similar changes to UP later in the day. This is possibly related to variations in other symptoms such as mood, or diurnal or circadian fluctuations. Naturally, recordings from six acutely ill inpatients in a mixed state cannot fully answer whether activity patterns in mixed episodes more closely resemble those of mania or depression, but this study does indicate that any exploration of actigraphy patterns should not assume mixed states can be combined with mania cases to form a single group. Also, as the six mixed cases could be distinguished from BD depression and UP in the morning and evening, respectively, it does lend some credence to the value of our methodology of detailed activity analysis, rather than reliance on mean counts per minute over 24 h. We can only suggest points of interest for future research due to the few cases included here. Accordingly, both linear and non-linear activity measures and time of day should be considered in further exploration of mixed state profiles, as well as correlations between diurnal changes in activity and mood.

There are several limitations to this study of activity patterns across subtypes and phases of mood disorders and course of day. The size of the subgroups reflects admission rates for these different clinical presentations during the 7-month recruitment period for the study, but led to uneven group sizes. The size of the UP group is adequate in comparison to other studies [5], but the individual BD groups (although about average in comparison to other actigraphy studies in clinical samples) are small, especially mixed states and BD depression. Also, we did not control for several potential confounders, most notably severity or duration of current illness episode, and there was no information on body mass index (BMI), past psychiatric history, comorbidity, family history or treatment. However, these limitations apply across all our subgroups and are likely to have a stronger confounding effect in studies that compare clinical cases to healthy controls than in studies that use a case vs. case design. Some of the factors related to the hospitalization, particularly medication, may have an important influence on the differences in activity patterns and could both explain or disguise true differences between subgroups. Finally, the actigraphy monitoring lasted for only 24 h, which undermines the generalizability of our findings and prevents assessment of day-to-day variations, which is another interesting phenomenon in mood disorders [12]. As we were aware of these limitations from the outset, we tried to focus on a small number of carefully selected parameters and to limit our statistical tests to the minimum required to ensure the pilot study offered a viable first step in exploring activity in inpatients with mood disorders.

## Conclusions

The current study provides novel findings by comparing UP and BD depression, by including mixed states as a separate group, by analyzing variability and complexity parameters and by comparing time series of morning and evening periods of continuous activity between and within groups. It is important to keep the preliminary nature of the study in mind, but the findings indicate that mood disorder subgroups can be differentiated by studying variability in activity on several temporal scales. Further research that compares diurnal and circadian variation in activity profiles in larger samples could aid in separating UP and BD depression and/or phases of BD, and provide further insight into the classification of mixed states. In a larger perspective, while we are not yet at the stage of using objective monitoring technologies to differentiate between subtypes of mood disorders or illness phases in clinical settings, such approaches are likely to be increasingly important in establishing reliable and valid diagnostic subtypes in future research. They also offer the prospect of combining clinical observation and actigraphy to improve our understanding of the phenomenology of UP and BD and the clinicopathological boundaries between subtypes of mood disorders.

## Abbreviations

BD: Bipolar disorder
BMI: Body mass index
Df: Degrees of freedom
DSM-5: The Diagnostic and Statistical Manual of Mental Disorders, 5th edition
ICD-10: International Classification of Diseases, 10th revision
RMSSD: Root mean squared successive difference
SD: Standard deviation
SPSS: Statistical Package for the Social Sciences
UP: Unipolar
WHO: World Health Organization
